# Polymer conjugated graphene-oxide nanoparticles impair nuclear DNA and Topoisomerase I in cancer†

**DOI:** 10.1039/c9na00617f

**Published:** 2019-11-06

**Authors:** Aditi Nandi, Chandramouli Ghosh, Sudipta Basu

**Affiliations:** Department of Chemistry, Indian Institute of Science Education and Research (IISER)-Pune Dr Homi Bhabha Road, Pashan Pune 411008 Maharashtra India; Discipline of Chemistry, Indian Institute of Technology (IIT)-Gandhinagar Palaj Gandhinagar Gujarat 382355 India sudipta.basu@iitgn.ac.in

## Abstract

Cancer chemotherapy had been dominated by the use of small molecule DNA damaging drugs. Eventually, the emergence of DNA damage repair machinery in cancer cells has led to combination therapy with the DNA topology controlling enzyme, topoisomerase I inhibitor along with DNA impairing agents. However, integrating multiple drugs having diverse water solubility and hence bio-distribution effectively for cancer treatment remains a significant challenge, which can be addressed by using suitable nano-scale materials. Herein, we have chemically conjugated graphene oxide (GO) with biocompatible and hydrophilic polymers [polyethylene glycol (PEG) and ethylene-diamine modified poly-isobutylene-maleic anhydride (PMA-ED)], which can encompass highly hydrophobic topoisomerase I inhibitor, SN38. Interestingly, these sheet structured GO-polymer-SN38 composites self-assembled into spherical nanoparticles in water after complexing with a hydrophilic DNA damaging drug, cisplatin. These nanoparticles showed much improved colloidal stability in water compared to their drug-loaded non-polymeric counterpart. These SN38 and cisplatin laden GO-polymer nanoparticles were taken up by HeLa cancer cells through clathrin-dependent endocytosis to home into lysosomes within 6 h, as confirmed by confocal microscopy. A combination of gel electrophoresis, flow cytometry, and fluorescence microscopy showed that these nanoparticles damaged nuclear DNA and induced topoisomerase I inhibition leading to apoptosis and finally improved HeLa cell death. These self-assembled GO-polymer nanoparticles can be used for strategic impairment of multiple cellular targets involving hydrophobic and hydrophilic drugs for effective combination therapy.

## Abstract 2

Polymer modified self-assembled graphene oxide nanoparticles are developed for targeting cancer cells.

## Graphical abstract

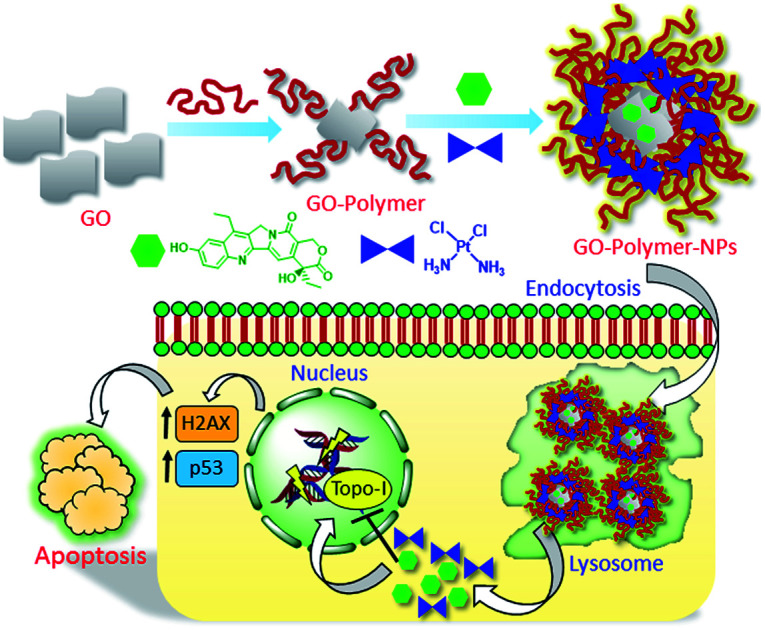

## Introduction

Cancer is a multifactorial disease with complex origin and development.^1^ Cellular DNA plays a critical role in cancer progression.^2^ Nuclear DNA controls important regulatory processes such as replication and transcription which directly affect cellular proliferation, metabolism, gene activation/suppression, and cell cycle management. Hence, DNA was established as the primary target and interaction site of the majority of chemotherapeutic drugs.^3^ These drugs target rapidly proliferating cancer cells by directly or indirectly damaging nuclear DNA leading to the eradication of cancer cells.^4–6^ While chemotherapy has been successful in the treatment of various types of cancer, its effectiveness is often hampered in the long run by the onset of toxic side effects and drug resistance.^7–9^ Thus identification of new suitable targets will help enhance the therapeutic outcome of the DNA damaging chemotherapeutic drugs.^10–12^

In recent years, topoisomerases have been proven to be viable therapeutic targets for anticancer therapy because of their essential role in several biological mechanisms.^13–16^ These ubiquitous enzymes belong to a protein superfamily responsible for maintaining DNA topology by relaxing the DNA supercoil generated during DNA replication, DNA transcription, chromosomal condensation, and segregation.^15^ Particularly, topoisomerase I (TOP1) resolves the torsional stress in DNA by introducing a reversible single-strand break which allows the rotation of the cleaved DNA strand around the intact strand.^17–19^ The transient reversible cleavable complex of TOP1 and the DNA strand has been a vulnerable target of various novel antitumor drugs, including camptothecin and its analogues. Although camptothecin and its derivatives are effective TOP1 inhibitors, their use is narrow due to dose-limiting toxicity and erratic bio-distribution.^20–23^ Nonetheless, the synergistic effect of TOP1 inhibitors with DNA damaging drugs has been in use in clinics for different malignancies.^24–26^ However, this combination therapy faces hurdles of augmented toxicity and uncontrolled bio-distribution. Nanotechnology-based platforms can solve this bottleneck.

The inclusion of nanotechnology in cancer therapy led to the discovery and development of novel nano-materials with advantageous properties like enhanced drug loading, controlled release, increased tumor accumulation and reduced side-effects for biomedical applications.^27–31^ In this context, graphene oxide (GO) has garnered significant interest owing to its biocompatibility and plethora of applications especially in cancer treatment for the delivery of therapeutics (drugs, genes, and proteins).^32–42^ The superiority of GO stems from its unique 2-dimensional structure combined with oxygen-rich functionalities (epoxide, carboxylic acids, and alcohols) present for tagging therapeutic entities. However, chemical conjugation of multiple drugs with hydrophilic functionalities in GO hugely compromises the solubility of the GO–drug conjugates for further biological applications. This challenge could be overcome by conjugating GO with biocompatible and hydrophilic polymers for trafficking hydrophobic therapeutic payload into cancer cells.^43–47^

Towards this end, herein, we have synthesized GO-polymer conjugates from polyethylene glycol (PEG) and poly isobutylene-maleic acid-ethylenediamine (PMA-ED) followed by incorporating SN38 (topoisomerase I inhibitor) and cisplatin (DNA damaging drug) through π–π interactions and chemical conjugation respectively. These dual drug-laden GO-polymer conjugates remarkably self-assembled into spherical nanoscale particles (GO-PEG-NPs and GO-PMA-NPs) with enhanced water dispersibility. These GO-polymer NPs were taken up by HeLa cells *via* clathrin-controlled internalization into lysosomes. Inside the cancer cells, GO-polymer NPs inhibited TOP1 concomitantly with nuclear DNA impairment to induce programmed cell death (apoptosis). These polymer conjugated self-assembled nanoscale particles with higher aqueous dispersibility can be further explored as a flexible platform to load multiple drug combinations having specific targets in the cellular milieu, thus enhancing their therapeutic efficacy for future combination therapy.

## Result and discussion

### Synthesis of GO-polymer conjugates

**Scheme 1:**
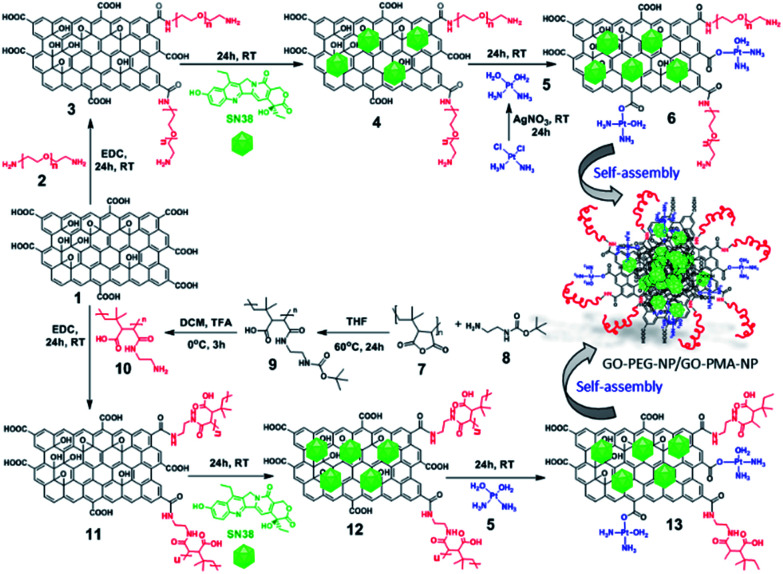
Synthetic scheme of GO-PEG-SN38-CDDP and GO-PMA-SN38-CDDP conjugates.

**Fig. 1:**
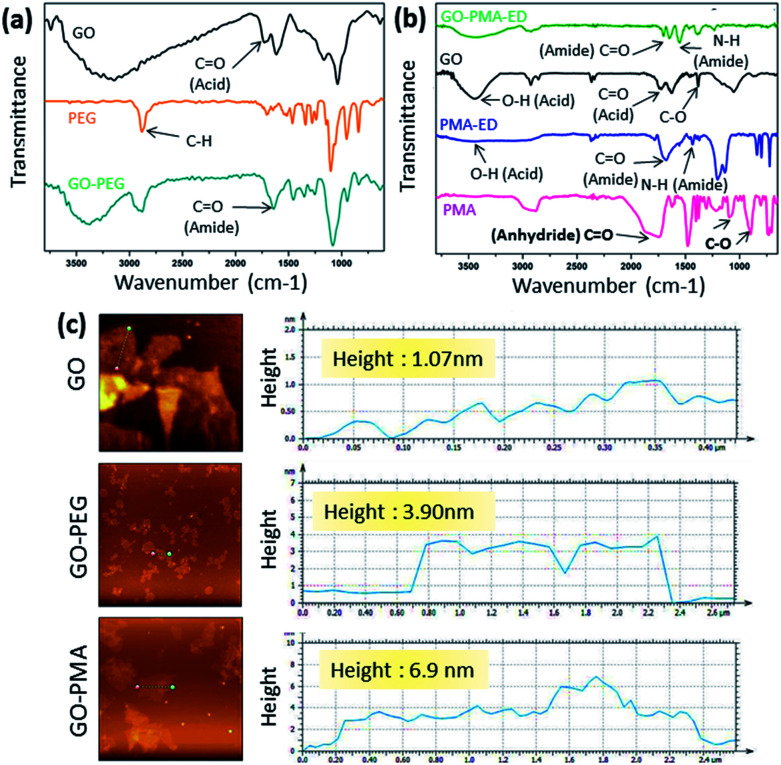
(a and b) FT-IR spectra of GO-PEG and GO-PMA-ED conjugates. (c) AFM images and height profile of GO, GO-PEG, and GO-PMA-ED conjugates.

To synthesize GO-polymer conjugates, we first chemically reacted GO (1) with (polyethylene)bis(amine) (2) in a 1 : 5 weight ratio in the presence of EDC as a coupling agent to obtain the GO-PEG conjugate (3) through amide linkage (Scheme 1). The conjugation of PEG with GO was confirmed by FT-IR spectroscopy. The FT-IR spectra of GO displayed the signature peaks at 1720 cm^−1^ (C=O stretching), a broad peak at 3400 cm^−1^ (O–H stretching), and a peak at 1050 cm^−1^ (C–O stretching). After conjugation with PEG through amide linkage, GO-PEG showed a new peak at 1640 cm^−1^ for C=O stretching in the amide bond (Fig. 1a). We have also used another biocompatible polymer poly(isobutylene-*alt*-maleic anhydride) (PMA) to conjugate with GO.^48^ For this, we first reacted PMA (7) with *N*-Boc-protected ethylenediamine (8) to open up the anhydride linkage in PMA to form the PMA-Boc-ED conjugate (9) in the presence of THF as the solvent at 60 °C for 24 h (Scheme 1). We further de-protected the ethylenediamine in the presence of trifluoroacetic acid (TFA) in DCM at 0 °C for 24 h to obtain the PMA-ED conjugate (10). The PMA-ED conjugate was characterized by FT-IR, which revealed characteristic peaks at 1670 cm^−1^ and 1560 cm^−1^ for C=O stretching and N–H bending modes in the amide functionality respectively (Fig. 1b). We also characterized PMA-Boc-ED (9) and PMA-ED (10) polymers by ^1^H NMR spectroscopy (Fig. S1 and S2, ESI†). We then conjugated PMA-ED with GO using EDC as a coupling agent at room temperature for 24 h to obtain the GO-PMA conjugate (11). The retention of C=O stretching and N–H bending peaks in FT-IR confirmed the formation of the amide bond in the GO-PMA-ED conjugate (Fig. 1b). We visualized the morphology and calculated the layer thickness of GO-PEG and GO-PMA-ED conjugates by atomic force microscopy (AFM). The AFM analysis confirmed the 2D-sheet like morphology of GO-PEG and GO-PMA-ED similar to that of pristine GO (Fig. 1c). The height calculation revealed the increase in height to 3.9 nm and 6.9 nm for GO-PEG and GO-PMA-ED compared to 1.07 nm for unmodified GO (Fig. 1c). This considerable increase in height also indicated polymer conjugation on the GO surface.

### Synthesis of drug loaded GO-polymer nanoparticles

**Fig. 2:**
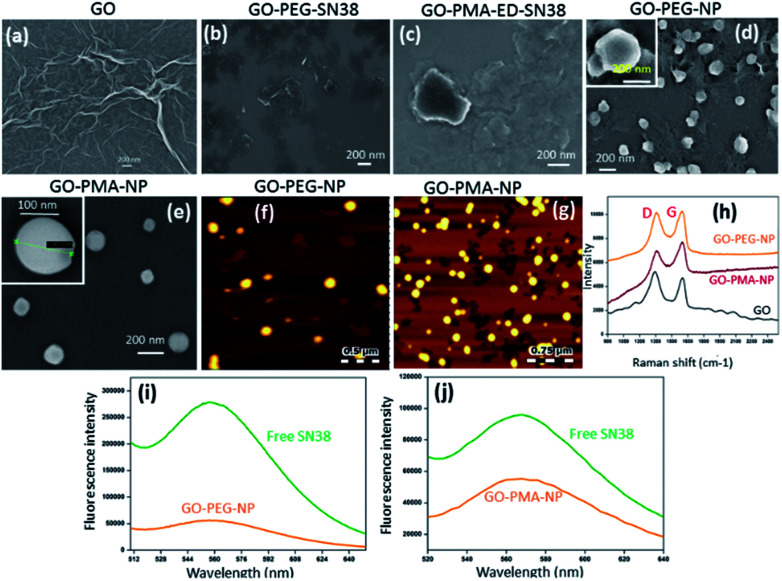
(a–e) FESEM images of GO, GO-PEG-SN38, GO-PMA-ED-SN38, GO-PEG-NPs, and GO-PMA-NPs, respectively. (f and g) AFM images of GO-PEG-NPs and GO-PMA-NPs, respectively. (h) Single-particle resonance Raman spectra of GO, GO-PEG-NPs, and GO-PMA-NPs. (i and j) Fluorescence emission spectra of GO-PEG-NPs and GO-PMA-NPs respectively compared to free SN38.

For targeting topoisomerase I in the nucleus, we chose SN38, an active metabolite of camptothecin. However, low water solubility and side effects like neutropenia and anemia limited its use in clinics.^49^ Hence, we used SN38 to increase its water solubility through our GO-polymer conjugates. We reacted SN38 with GO-PEG (3) and GO-PMA-ED (11) in a water/DMSO mixture at room temperature for 24 h to stack it on the GO surface by π–π interactions to obtain GO-PEG-SN38 (4) and GO-PMA-ED-SN38 (12) composites (Scheme 1). The morphology of GO-PEG-SN38 and GO-PMA-ED-SN38 was visualized by FESEM. The FESEM images clearly showed that after the stacking of SN38 on the GO surface, the composites 4 and 11 retained their 2D-sheet structure similar to GO (Fig. 2a–c).

Furthermore, for simultaneous targeting of topoisomerase I along with nuclear DNA, we would like to introduce cisplatin as a DNA damaging drug with SN38. Moreover, cisplatin showed a synergistic effect in the presence of SN38 in different cancer cells.^24–26^ Hence, we further reacted [(NH_3_)_2_Pt(OH_2_)_2_]^+^ obtained from cisplatin (CDDP) after reacting with silver nitrate, with GO-PEG-SN38 (4) and GO-PMA-ED-SN38 (12) composites at room temperature for 24 h to obtain GO-PEG-SN38-CDDP (6) and GO-PMA-ED-SN38-CDDP (13) composites (Scheme 1). We visualized the morphology of the dual drug-loaded composite 6 and 13 by FESEM and AFM. To our surprise, in the presence of cisplatin, both the composites self-assembled into 3D-spherical nanoparticles with a diameter less than 200 nm from 2D-sheet like structures to form GO-PEG-NPs and GO-PMA-NPs (Fig. 2d–g and S3, ESI†). This remarkable transformation in morphology is in accordance with our previous observations.^50^ We confirmed the presence of the GO moiety in the nanoparticles by resonance Raman spectroscopy. On particle resonance Raman spectra clearly showed that both GO-PEG-NPs and GO-PMA-NPs contained characteristic D and G bands of GO at 1350 cm^−1^ and 1590 cm^−1^ respectively (Fig. 2h). Successful stacking of SN38 on the self-assembled nanoparticles by π–π interactions was evaluated by the remarkable quenching in the fluorescence emission intensity of SN38 at *λ*_max_ = 560 nm compared to free SN38 in the same concentration (Fig. 2i and j). To validate the presence of cisplatin, energy dispersive X-ray spectroscopy (EDX) was carried out on a single particle, which showed that ∼19.6 weight% and 10.2 weight% of Pt were present in GO-PEG-NPs and GO-PMA-NPs respectively (Fig. S4, ESI†). Confirmation of the co-loading of cisplatin and SN38 along with the determination of their loading was carried out by UV-vis spectroscopy. From the absorbance *versus* concentration calibration graph of SN38 (*λ*_max_ = 387 nm) and cisplatin (*λ*_max_ = 706 nm) (Fig. S5a and b, ESI†), the loading of SN38 and cisplatin was found to be 1364.3 μM (535 μg mL^−1^) and 1100 μM (330 μg mL^−1^) respectively in GO-PEG-NPs (Fig. S5c, ESI†). On the other hand, the loading of SN38 and cisplatin was found to be 1321 μM (518 μg mL^−1^) and 1290 μM (387 μg mL^−1^) respectively in GO-PMA-NPs (Fig. S5d, ESI†). Finally, we studied the time-dependent colloidal stability of GO-PEG-NPs and GO-PMA-NPs in water. The dispersibility images clearly showed that both the nanoparticles demonstrated enhanced colloidal stability over 140 minutes compared to non-polymer modified GO-SN38-CDDP-NPs which agglomerated within 10 minutes (Fig. S6, ESI†).

### Cellular internalization

**Fig. 3:**
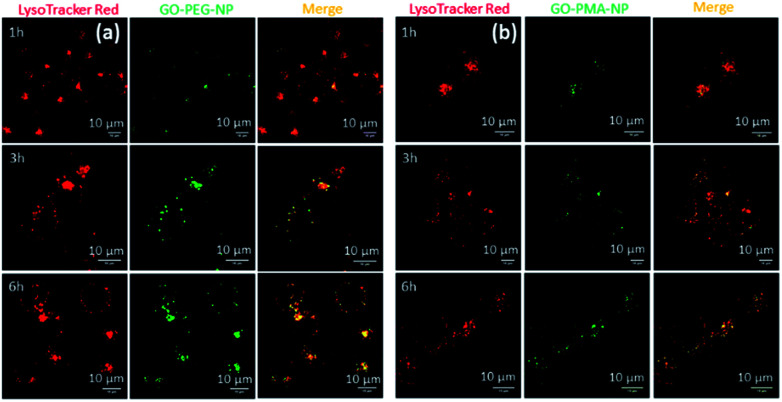
(a and b) Confocal laser scanning microscopy images of HeLa cells after treatment with GO-PEG-NPs and GO-PMA-NPs (green fluorescence) in a time-dependent manner. Lysosomes were stained with LysoTracker Red DND-99 dye. Scale bar = 10 μm.

After successfully engineering dual drug-loaded GO-polymer-NPs, we studied their effects on cancer cells. We hypothesized that these nanoparticles would be internalized into cancer cells and home into lysosomes.^50^ To validate this hypothesis, we incubated HeLa cervical cancer cells with green fluorescent GO-PEG-NPs and GO-PMA-NPs at 1 h, 3 h, and 6 h. The lysosomes were stained with LysoTracker Red DND-99, and the cells were viewed under a confocal microscope. The fluorescence microscopy images clearly showed the cellular uptake and time-dependent lysosomal colocalization of green fluorescent GO-polymer-NPs into red fluorescently labeled lysosomes from the gradual increase of the yellow intensity due to overlapping of green and red signals at 1 h, 3 h, and 6 h (Fig. 3a and b). Quantification of the confocal images through Mander's and Pearson's coefficients for the extent of overlapping of red and green fluorescence signals confirmed the time-dependent localization of the nanoparticles into lysosomes with 15%, 25% and 45% colocalization volume for GO-PEG-NPs and 11%, 23% and 37% for GO-PMA-NPs (Tables S1 and S2, ESI†).

**Fig. 4:**
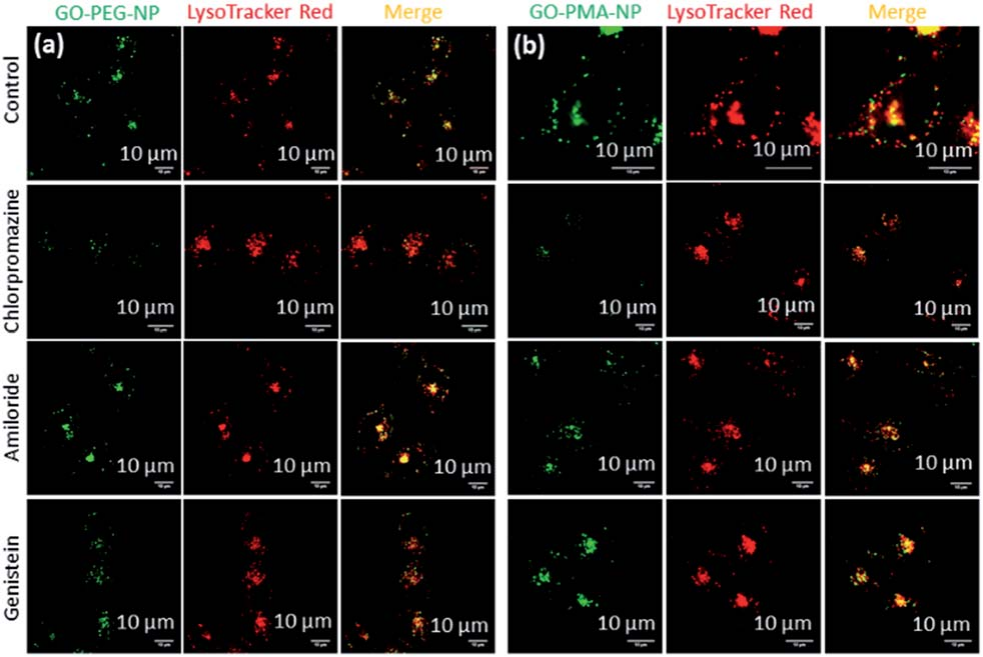
(a and b) Confocal laser scanning microscopy images of HeLa cells after treatment with endocytosis inhibitors (chlorpromazine, amiloride, and genistein) followed by incubation with GO-PEG-NPs and GO-PMA-NPs (green fluorescence). Lysosomes were stained with LysoTracker Red DND-99 dye. Scale bar = 10 μm.

The engulfment of the nano-scale particles by cells through different endocytosis pathways varies depending on the size and shape of the particles. To determine the mechanism of endocytosis, HeLa cells were treated with endocytosis inhibitors (chlorpromazine, genistein, and amiloride) for 45 min, followed by incubation with GO-PEG-NPs and GO-PMA-NPs (green fluorescence) for 2 h. Lysosomes were stained with LysoTracker Red DND-99. Visualization by confocal microscopy revealed that cells treated with genistein and amiloride showed no significant change in the cellular uptake and lysosomal homing of both the nanoparticles compared to no-inhibitor treated cancer cells (Fig. 4a and b). On the contrary, a notable reduction in the co-localization (yellow signal intensity) was observed for cells treated with chlorpromazine and incubated with GO-PEG-NPs and GO-PMA-NPs. The image-based quantification for a co-localization volume of 12% (chlorpromazine), 41% (amiloride), 27% (genistein) and 39% (control) for GO-PEG-NPs supported the imaging data (Table S3, ESI†). Similarly, only 16% co-localization was found for chlorpromazine treated cells compared to 35%, 31%, and 41% for amiloride, genistein, and control cells for GO-PMA-NPs (Table S4, ESI†).

Post localization into acidic lysosomes, GO-PEG-NPs and GO-PMA-NPs should release their dual drugs for effective DNA damage and topoisomerase I inhibition. To study the release of SN38 and cisplatin, both the nanoparticles were incubated in an acidic buffer (pH = 5.5, lysosome mimic) over 72 h. The release was monitored and quantified at different time intervals by UV-vis spectroscopy. It was found that GO-PEG-NPs released 46% of SN38 and 36% of cisplatin after 72 h, respectively (Fig. S7a, ESI†). On the other hand, GO-PMA-NPs released 45% and 36% of SN38 and cisplatin, respectively (Fig. S7b, ESI†). Higher release of SN38 compared to cisplatin can be attributed to the weaker π–π stacking interaction between GO and SN38 as compared to the stronger coordination linkage between cisplatin and the COOH group of GO. Alternatively, quantification of dual drug release at physiological pH of 7.4 revealed only 19.5% of SN38 and 10% of cisplatin from GO-PEG-NPs (Fig. S7, ESI†) and 20% of SN38 and 11% of cisplatin from GO-PMA-NPs were released at 72 h. We anticipate that at lower pH (pH = 5.5), SN38 will be protonated, leading to the weakening of hydrophobic and π-stacking interactions with the GO moiety compared to physiological pH. This could be the potential explanation of the higher release of SN38 from the polymer-GO-NPs at pH = 5.5 compared to pH = 7.4. On the other hand, the plausible mechanism of higher release of cisplatin from GO-polymer-NPs involves efficient breaking of the Pt–carboxylato bonds in acidic medium (pH = 5.5) compared to physiological pH.^50^ The drug release profile at pH = 5.5 and 7.4 indicated that the nanoparticles are expected to release the chemotherapeutic payload better while residing in lysosomes inside the cancer cells, rather than in blood circulation, which is essential for efficient targeting of tumor tissues and not non-cancerous tissues through passive targeting.

### DNA cleavage and topoisomerase I inhibition

**Fig. 5:**
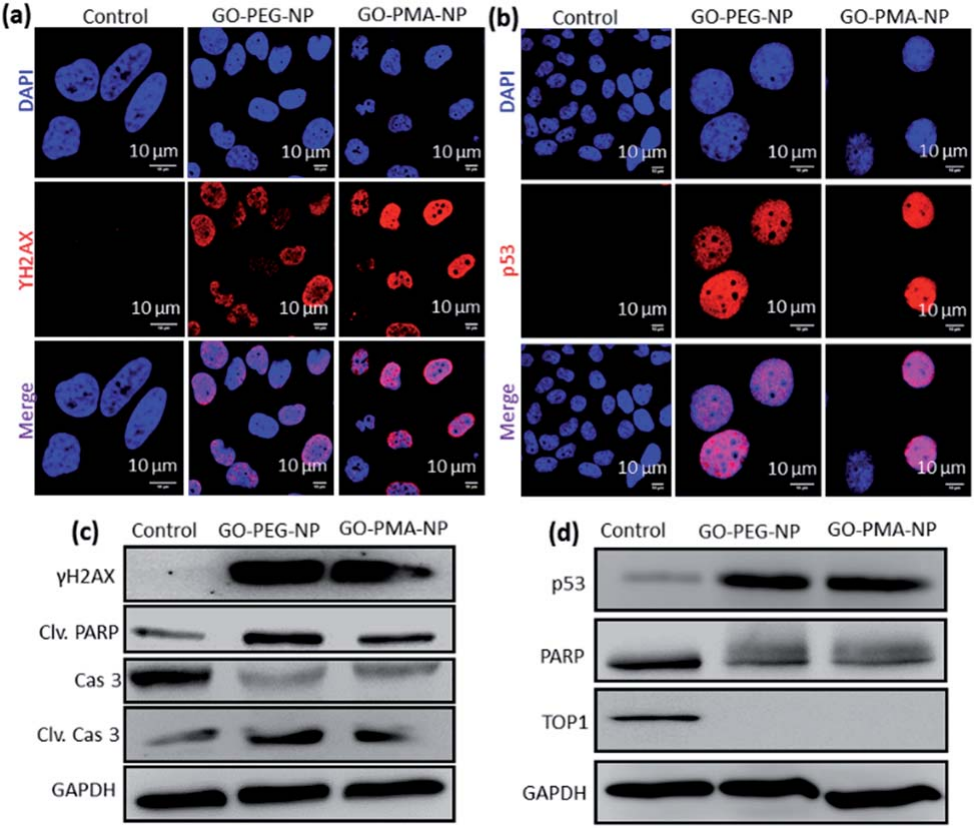
(a and b) Confocal laser scanning microscopy images of HeLa cells after treatment with GO-PEG-NPs and GO-PMA-NPs for 24 h followed by incubation with primary antibodies specific for ϒH2AX and p53 which were further stained with a secondary antibody tagged with Alexa Fluor 549 (red fluorescence). Nuclei were stained with the blue fluorescent dye DAPI. Scale bar = 10 μm. (c and d) Western blot images of ϒH2AX, p53, PARP, cleaved-PARP, topoisomerase-I, caspase-3 and cleaved caspase-3 in HeLa cells after treatment with GO-PEG-NPs and GO-PMA-NPs for 24 h.

We hypothesized that the lysosomal release of cisplatin and SN38 from GO-polymer-NPs could lead to DNA damage along with topoisomerase I inhibition. For determining the DNA damaging ability, we estimated the expression of ϒH2AX and p53 by immunofluorescence, which are DNA damage biomarkers. HeLa cells were incubated with GO-PEG-NPs and GO-PMA-NPs for 24 h followed by treatment with anti-ϒH2AX and anti-p53 primary antibodies and red fluorescent Alexa Fluor 549-tagged secondary antibody. The nucleus of the treated cells was stained with DAPI (blue) after which the expression of ϒH2AX and p53 was observed by confocal microscopy. Fig. 5a and b display the increased expression of ϒH2AX and p53 through the higher red fluorescence signal indicating DNA damage as compared to non-treated control cells, which showed negligible ϒH2AX and p53 expression. Moreover, the overlapping of red and green fluorescence signals leading to a purple signal confirmed that both the nanoparticles induced nuclear DNA damage. Fluorescence signal-based quantification also revealed that GO-PEG-NPs and GO-PMA-NPs caused 5 fold and 6.4 fold increase in ϒH2AX expression (Fig. S8a, ESI†). On the other hand, GO-PEG-NPs and GO-PMA-NPs increased the expression of p53 by 4 fold and 5 fold, respectively compared to control cells (Fig. S8b, ESI†).

Moreover, western blot analysis for the expression of ϒH2AX asserted the nuclear DNA damage of HeLa cells after 24 h incubation with the nanoparticles. 2.3 fold and 1.8 fold amplification of ϒH2AX expression for GO-PEG-NPs and GO-PMA-NPs was evident from western blot images as compared to control cells (Fig. 5c and S9a, ESI†). Also, the expression of p53 after the treatment with nanoparticles for 24 h was evaluated by western blot. Fig. 5d clearly demonstrated that GO-PEG-NPs and GO-PMA-NPs increased the expression of p53 by 6.3 fold and 6.4 fold, respectively (Fig. S9b, ESI†). As a response to DNA damage, the cellular repair machinery through the poly-ADP-ribose (PARP) family of proteins gets triggered in cells.^51,52^ Assessment of the expression of PARP post treatment with GO-polymer-NPs by western blot evidently showed the reduction of PARP expression by 1.6 fold and 1.4 fold respectively (Fig. 5d and S9c, ESI†). The downregulation of PARP expression as compared to un-treated cells can be attributed to its cleavage because of DNA damage. The subsequent 3 fold and 2 fold increase in expression of cleaved PARP by GO-PEG-NPs and GO-PMA-NPs respectively was also evaluated by western blot (Fig. 5c and S9d, ESI†).

We also evaluated the inhibition of topoisomerase I induced by GO-polymer-NPs by western blot analysis. To account for topoisomerase I inhibition due to SN38, HeLa cells were treated with GO-PEG-NPs and GO-PMA-NPs for 24 h followed by gel electrophoresis of the whole-cell proteins. The western blot image and quantification exhibited that GO-PEG-NPs and GO-PMA-NPs down-regulated the expression of topoisomerase I by 66 fold and 19 fold, respectively (Fig. 5d and S9e, ESI†). The confocal images and western blot analysis confirmed that GO-polymer-NPs damaged nuclear DNA and inhibited topoisomerase I in HeLa cells.

### Apoptosis

**Fig. 6:**
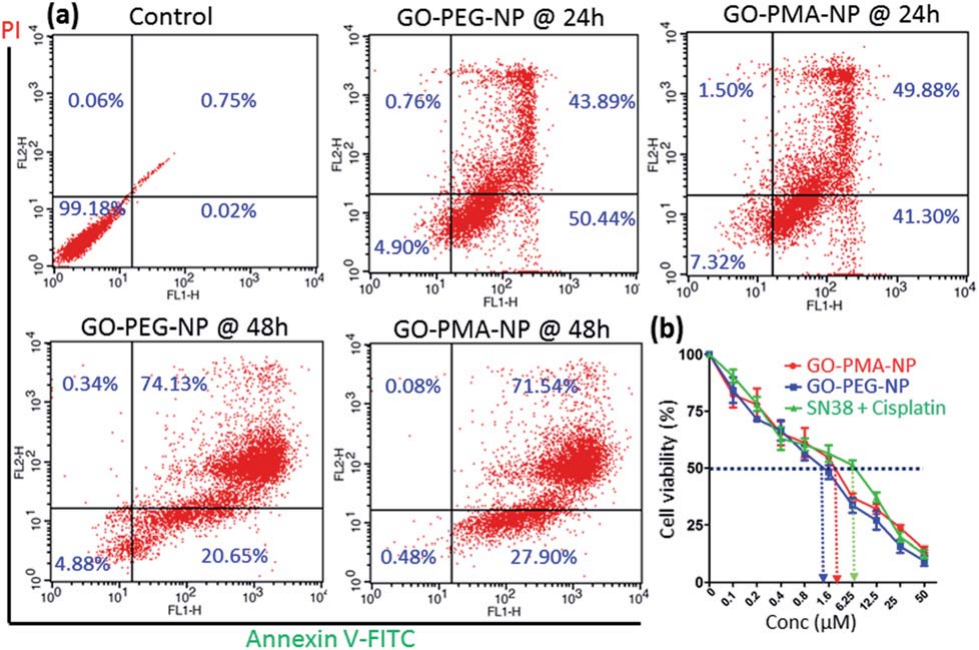
(a) Flow cytometry analysis of apoptosis in HeLa cells after treatment with GO-PEG-NPs and GO-PMA-NPs for 24 h and 48 h. The apoptotic and necrotic cells were stained with green fluorescent Annexin V-FITC and PI (red fluorescence). (b) Cell viability by MTT assay in HeLa cells after treatment with GO-PEG-NPs and GO-PMA-NPs at 48 h post incubation.

Evading apoptosis is one of the most important hallmarks of cancer cells.^53^ Hence, we estimated the apoptosis-inducing ability of GO-polymer-NPs by flow cytometry. HeLa cells were treated with the GO-PEG-NPs and GO-PMA-NPs for 24 h and 48 h followed by co-staining with Annexin V-FITC (binds to the surface phosphatidylserine of apoptotic cells) and PI (binds to the DNA of apoptotic and necrotic cells). From the flow cytometry analysis (Fig. 6a) we observed that in comparison to non-treated control cells, HeLa cells treated with GO-PEG-NPs for 24 h showed 50.45% and 43.89% cells in the early and late apoptotic stages respectively. After 48 h of incubation, the percentage of HeLa cells undergoing late apoptosis increased to 74.13% with 20.65% cells in the early apoptotic stage. Similarly, after 24 h of incubation, GO-PMA-NPs prompted 41.30% and 49.88% cells in early and late apoptosis, whereas 27.90% and 71.54% cells were in the early and late apoptotic state at 48 h post incubation.

DNA damage-topoisomerase I inhibition-induced apoptosis was further confirmed by cleavage of caspase 3 (an important inducer of apoptosis) using western blot analysis.^54^ The decrease in the expression of caspase 3 by 3.0 fold and 2.3 fold (Fig. 5c and S10a, ESI†) and the corresponding increase in the expression of cleaved caspase 3 by 3.0 fold and 1.8 fold (Fig. 5c and S10b, ESI†) compared to control cells displayed the ability of GO-PEG-NPs and GO-PMA-NPs respectively to successfully induce apoptosis in cervical cancer HeLa cells.

Finally, since the as-synthesized GO-polymer based nanoparticles effectively caused topoisomerase I inhibition, DNA damage, and induced apoptosis in HeLa cells, we evaluated the cancer cell killing efficacy by MTT assay. GO-PEG-NPs and GO-PMA-NPs were incubated with HeLa cells for 48 h in a dose-dependent manner. As a control, we treated cells with the free drug combination of SN38 and cisplatin in the same ratio as that present in the respective nanoparticles. MTT data revealed that GO-PEG-NPs and GO-PMA-NPs (Fig. 6b) killed 50% cells (IC_50_) at a much lower concentration of 1.5 μM and 2.5 μM as compared to the free drug cocktail which displayed a much higher IC_50_ value of 6.25 μM. Hence, from these flow cytometry and cell viability assays, it was confirmed that GO-polymer-NPs triggered apoptosis in HeLa cells, leading to cell death.

## Conclusions

In this current study, we have successfully designed polymer functionalized self-assembled graphene oxide (GO) spherical nanoparticles which can encompass SN38 (topoisomerase I inhibitor) and cisplatin (DNA damaging drug). We chemically modified pristine 2-dimensional GO sheets with hydrophilic polymers like PEG and PMA and self-assembled them into 3-dimensional spherical nanoparticles through cisplatin cross-linking. These as-formed GO-PEG-NPs and GO-PMA-NPs displayed enhanced aqueous colloidal stability, which is an important aspect of effective biomedical application of nano-scale materials. The average diameter of these nanoparticles was around 180 nm, which could facilitate their specific accumulation into cancer cells through the enhanced permeability and retention (EPR) effect. The GO-PEG-NPs and GO-PMA-NPs were taken up by HeLa cells through clathrin-induced endocytosis, into the acidic lysosomes within 6 h and triggered the release of SN38 and cisplatin as payloads. This nanoparticle-induced DNA damage and topoisomerase I inhibition prompted apoptosis in cancer cells which was confirmed by western blot and flow cytometry analysis. Furthermore, the GO-polymer-NPs demonstrated improved HeLa cell killing efficacy in comparison to the free drug cocktail. Thus, our strategy represents an improved design to increase the water dispersibility of GO nanoparticles and their potential usage in clinics for future combination chemotherapy.

## Conflicts of interest

There are no conflicts to declare.

## Supplementary Material

NA-001-C9NA00617F-s001
