# Supplementary material for: Polymer conjugated graphene-oxide nanoparticles impair nuclear DNA and Topoisomerase I in cancer
Source: Nanoscale Adv. 2019 Nov 6;1(12):4965–71. doi: 10.1039/c9na00617f (PMC9417292; doi:10.1039/c9na00617f)
Supplement: NA-001-C9NA00617F-s001 [file NA-001-C9NA00617F-s001.pdf]

Electronic Supplementary Material (ESI) for Nanoscale Advances.
This journal is © The Royal Society of Chemistry 2019

Supporting Information

Polymer Conjugated Graphene-Oxide Nanoparticles Impair Nuclear DNA and Topoisomerase I in Cancer

Aditi Nandi,[a] Sudipta Basu*[b]

a. Department of Chemistry, Indian Institute of Science Education and Research (IISER)-Pune, Dr. Homi Bhabha Road, Pashan, Pune, 411008, Maharashtra, India

b. Discipline of Chemistry, Indian Institute of Technology (IIT)-Gandhinagar, Palaj, Gandhinagar, Gujarat, 382355, India.

* Corresponding author. Email: Sudipta.basu@iitgn.ac.in

Experimental Section

Materials. Graphene oxide (4mg/ml), 1-Ethyl-3-(3-dimethylaminopropyl)carbodiimide hydrochloride (EDC), Poly(isobutylnene-alt-maleic anhydride) (average. M_w ~6000), Poly(ethyleneglycol)bis(amine) (average M_n ~3400), Triethylamine, silicon wafer for FESEM, 8 well LabTek chamber slides and solvents needed for synthesis were purchased from Sigma-Aldrich. Anticancer drug SN38 was bought from Selleck Chemicals and was used without any further purification. Gibco-DMEM (Dulbecco's Modified Eagle Meduim), fetal bovine serum (FBS), LysoTracker™ Green DND-99, were procured from ThermoFisher Scientific. 3-(4, 5-dimethylthiazol-2-yl)-2,5-diphenyltetrazolium bromide (MTT), Sodium Dodecyl Sulphate (SDS), 96 well plates, and 6 well plates were purchased from HiMedia. 15 mL and 50 mL graduated sterile centrifuge tubes and tissue culture flasks with filter cap sterile were purchased from Tarsons Product Pvt. Ltd. Annexin-V-FITC Staining Kit was purchased from Biolegend. Anti-PARP antibody-clone 7A10, anti-phospho-histone H2AX (Ser139) antibody- clone JBW301, GAPDH antibody, antigoat anti-mouse IgG antibody, (H+L) HRP conjugate and rabbit. HRP conjugates were obtained from Cell Signalling. The release kinetic data, drug loading, nanoparticle size and cell viability assay were plotted using Origin and GraphPad Prism software. The laser scanning confocal microscopy was performed by TCS Leica SP8 machine. FACS analysis was performed using BD FACS Calibur™ flow cytometer.

Synthesis of Poly (isobutylnene-alt-maleic anhydride)-ethylenediamine conjugate (10).

300 mg (1.94 mmol of monomer) of poly(isobutylne-alt-maleic anhydride) (**7**, Scheme 1) was placed in a round bottom flask and dissolved in 5mL anhydrous THF. 2.34 mmol N-Boc-ethylenediamine (**8**, Scheme 1) dissolved in 3 mL of THF was quickly injected, sonicated for a few seconds and then kept at 60 °C under vigorous stirring. For quantitative reaction of maleic anhydride with the primary amine, the reaction mixture was concentrated roughly up to one fifth of the original volume using rotavapor system under a reduced pressure after 3 hours of reaction. Further, the concentrated solution was left overnight at 60 °C under stirring conditions. Finally, THF was completely evaporated and the resultant polymer was washed with cold diethyether and dried under vacuum to yield a yellowish powder (**9**, Scheme 1).

The compound was further dissolved in 3 mL dicholoromethane (DCM) and kept in an ice bath, into which 1.5 mL of TFA was added dropwise. The reaction mixture was stirred at room temperature for 3h. Finally, the solvent was evaporated under vacuum and remaining viscous liquid was washed with cold diethyether to get poly (isobutylne-alt-maleic anhydride)-ehtylenediamine conjugate (**10**) which was dried under vacuum.

Yield = 80% over 2 steps.

Synthesis of GO-Peg-SN38-Cisplatin and GO-PMA-ED-SN38-Cisplatin composites:

Poly (ethyleneglycol) bis (amine) (PEG) and poly (isobutylne-alt-maleic anhydride)-ehtylenediamine (PIMA-ED) were covalently linked to graphene oxide through EDC coupling chemistry as per reports.[1] Briefly, graphene oxide (4 mg/ml, 250 µL) was dispersed in distilled water (2 mL) followed by addition of 5 mg poly (ethyleneglycol) bis (amine) (PEG) or 5 mg poly (isobutylne-alt-maleic anhydride)-ehtylenediamine (PMA-ED) and EDC·HCl (4 mg) for sonication at room temperature for 30 min. The solution was then kept for stirring at room temperature overnight. The product GO-PEG was dialyzed (MW cut off:1000D) against distilled water for 2 days whereas, GO-PMA-ED was dialyzed as well as centrifuged and washed with water to remove unreacted PMA-ED polymer to obtain GO-PEG (3) and GO-PMA-ED (11) conjugates.

Further, SN-38 was dissolved in minimum amount of DMSO and reacted with GO-PEG (3) and GO-PMA-ED (11) in a 1:0.5 weight ratio in water for 24h followed by dialysis against distilled water for 1 day to remove DMSO and also centrifugation to remove unbound SN38 to obtain GO-PEG-SN38 (4) and GO-PMA-ED-SN (12) composites.[2] The obtained GO-PEG-SN38 and GO-PMA-ED-SN38 were then reacted with aquated cisplatin (CDDP) (5mg/mL) in 1:5 weight ratio in water for 24 h at room temperature. Excess aquated cisplatin was removed by dialysis for 6-8 hrs against distilled water which yielded the GO-PEG-SN38-CDDP (6) and GO-PMA-ED-SN38-CDDP (13).

Characterization.

FT-IR: Fourier transform infrared (FTIR) spectroscopy was performed using a NICOLET 6700 FTIR from Thermo Scientific.

Estimation of size, shape and morphology by FESEM, and AFM: The morphology of GO-PEG-SN38-CDDP and GO-PIMA_Ed-SN38-CDDP nanoparticles was observed using field-emission scanning electron microscopy (FESEM) and atomic force microscopy, by spotting the samples on a silicon wafer and mica sheet respectively and imaged using Carl Zeiss Ultraplus scanning electron microscope at an operating voltage of 4KV and Nanowizard Atomic force microscope.[3]

Raman Spectroscopy: Raman spectra for GO-PEG-SN38-CDDP and GO-PMA-NP were recorded with Lab RAM HR 800 (Horiba scientific) instrument using laser excitation wavelength of 532 nm with 50X objective.

Quantification of drug loading in nanoparticles: Loading of the individual drugs SN38 (387nm), Cisplatin (707 nm) in GO-PEG-NP and GO-PMA-NP was estimated by UV-visible spectroscopy and the drug loading efficiency was calculated as[4]:

$$\text{Drug encapsulation efficiency (\%)} = \frac{\text{Amount of drug loaded in nanoparticle} \times 100}{\text{Total amount of drug used}}$$

Ratio of loading of SN38:CDDP in GO-PEG-SN38-CDDP is 1:0.8 and in GO-PMA-ED-SN38-CDDP is 1:0.97.

Fluorescence Spectroscopy. Steady state fluorescence of for fluorescent drug SN38 was recorded using a Flouromax-4 (HORIBA scientific, USA) the emission spectra for SN38 was recorder at λ_{max} = 560nm.

Confocal laser scanning microscopy (CLSM)

Cellular Internalisation: 2X10^4 were seeded in a labtek chamber and incubated overnight at 5% CO_2 at 37°C for attachment. Cells were then treated with green fluorescent GO-PEG-NP (SN38:CDDP = 2μg mL^{-1}: 1.22 μg ml^{-1}) and GO-PMA-NP (SN38:CDDP = 2μg mL^{-1}: 1.4μg ml^{-1}) for 1h, 3h, and 6h. After incubation, the cells were washed with phosphate saline buffer (PBS) and treated with Lysotracker Red DND 99 and visualised using Leica SP8 confocal microscope.[3]

Study of endocytosis pathway: 2X10^4 HeLa cells were allowed to attach in 8 well LabTek and then pre-incubated with inhibitors: Chlorpromazine (10 μg mL-1), 5-(N-ethyl-N-isopropyl)-Amiloride (1 mM) and Genistein (200 μM) for 30 min. After 30 min, media was removed, washed with PBS and replaced with fresh DMEM. GO-PEG-NP (SN38: CDDP = 2μg mL^{-1}: 1.22 μg ml^{-1}) and GO-PMA-NPs (SN38:CDDP = 2μg mL^{-1}: 1.4μg ml^{-1}) were added and incubated for 2 h. The cells were washed with phosphate saline buffer (PBS) stained with Lysotracker Red DND 99, followed by which they were imaged using Leica SP8 confocal microscope.

Detection of γH2AX by immunostaining: 5 x 10^5 cervical cancer (HeLa) cells were seeded on a coverslip and allowed to attach overnight in a 5% CO_2 incubator at 37°C. GO-PEG-NP (SN38: CDDP = 1.5 μM:1.2 μM) and GO-PMA-NP were added (SN38: CDDP = 2.5 μM: 2.4 μM) and incubated for 24h. Cells were then incubated in primary antibody solution (γH2AX, PARP in 1:100 dilution) led by incubation with Alexa Flour-594 conjugated secondary antibody (1:500 dilution). The slides were subjected to fluorescence imaging using Leica SP8 confocal microscope.[4]

Western Blot analysis: 1X10^6 HeLa cells were treated with GO-PEG-NP (SN38: CDDP = 1.5 μM: 1.2 μM) and GO-PMA-NP (SN38: CDDP = 2.5 μM: 2.4 μM) for 24 h, after which cells were lysed to obtain the protein lysate and separated using SDS-polyacrylamide gel electrophoresis (SDS-PAGE). The desired proteins were further treated with the respective primary and secondary antibodies and visualized by GE Healthcare Lifesciences ImageQuant LAS 4000 using Immobilon Western Chemiluminescent HRP Substrate.[3]

Apoptosis detection by flow cytometry: After 24 h treatment with GO-PEG-NP (SN38: CDDP = 1.5 μM: 1.2 μM) and GO-PMA-NP (SN38: CDDP = 2.5 μM: 2.4 μM) NP at their corresponding IC_{50} concentrations, HeLa- cervical cancer cells were washed trypsinised and stained with Annexin V-FITC and PI (Dead Cell Apoptosis Kit). The cells were analysed for apoptosis using BD AccuriTM C6 flow cytometer.[3]

Cell Viability Assay: $5x10^3$ HeLa cells were incubated with GO-PEG-NPs and GO-PMA-NP in a concentration dependent manner. The viable cells were estimated using MTT reagent as described previously.[3]

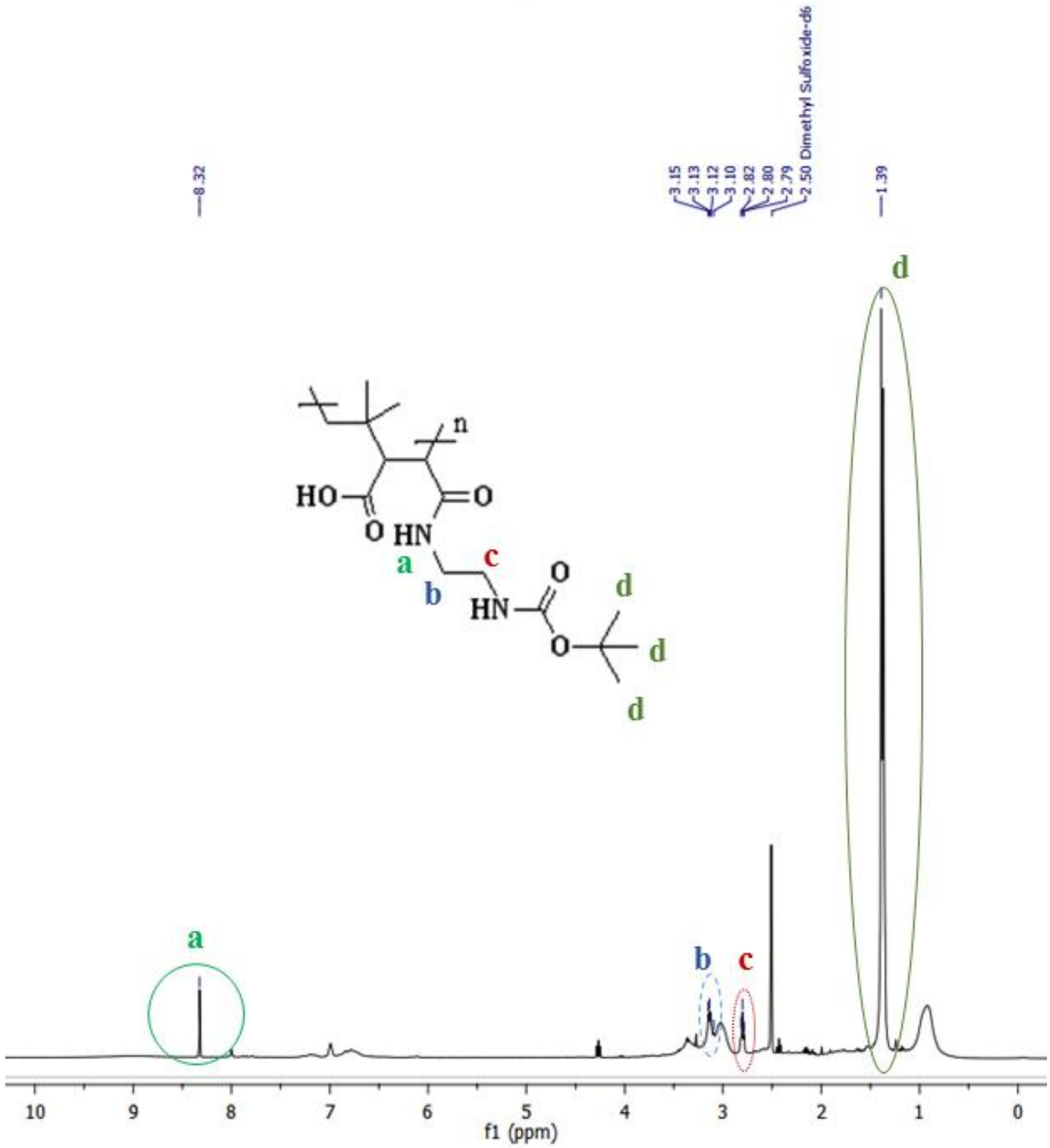

Fig. S1: ^{1}H NMR spectra of PMA-Boc-ED conjugate (9).

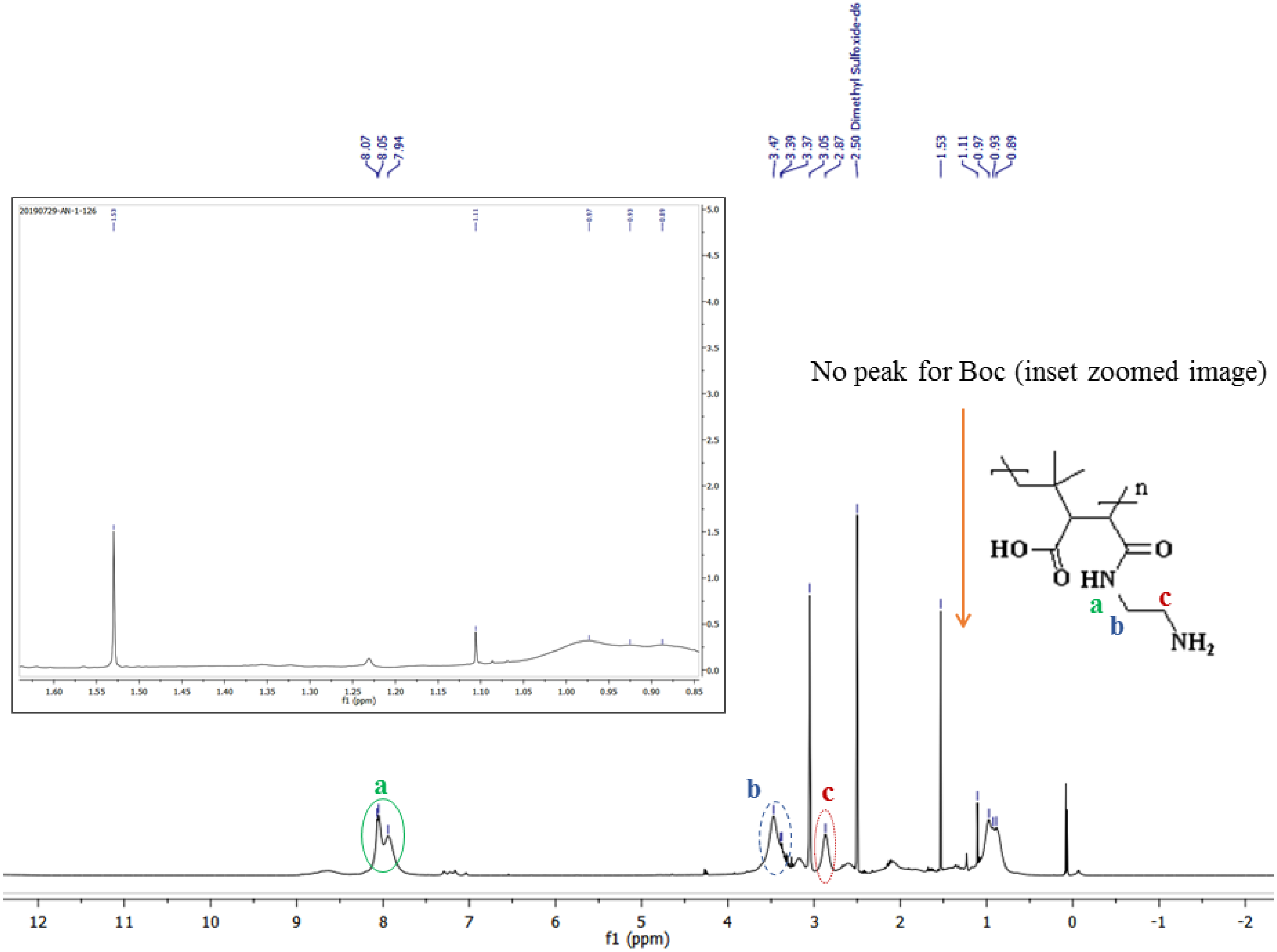

Fig. S2: ^{1}H NMR spectra of PMA-ED conjugate (10).

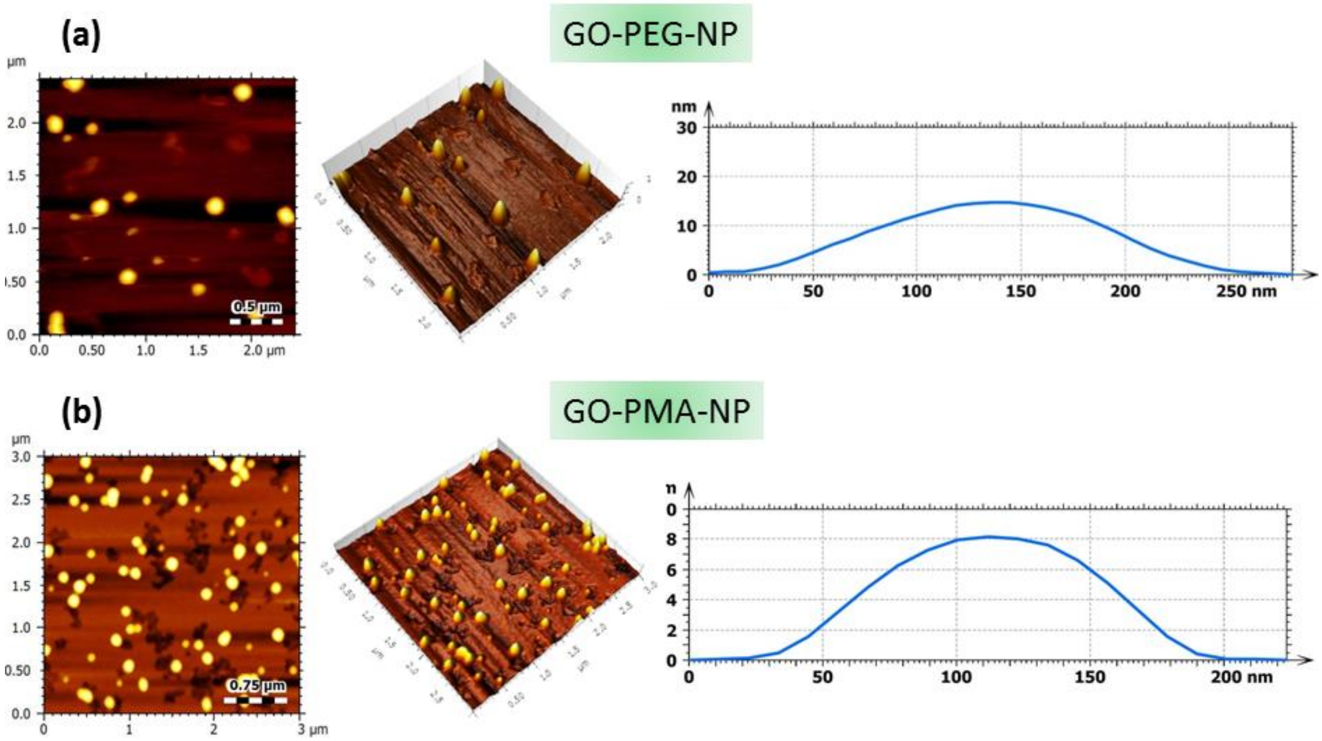

Fig. S3: (a,b) 2D, 3D and height plot of GO-PEG-NPs and GO-PMA-NPs respectively by AFM.

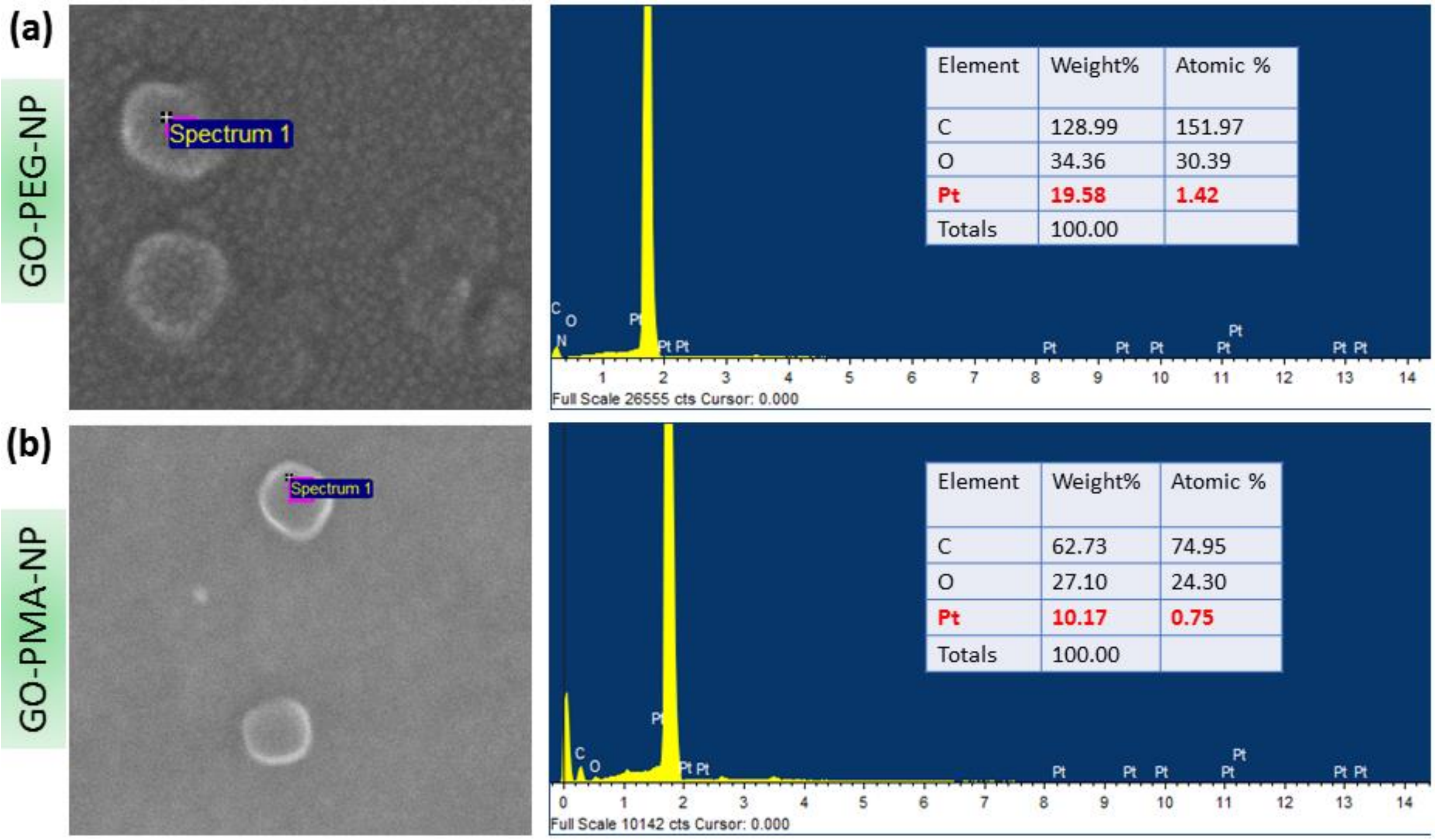

Fig. S4: (a,b) EDX spectra of GO-PEG-NPs and GO-PMA-NPs respectively to confirm the presence of cisplatin.

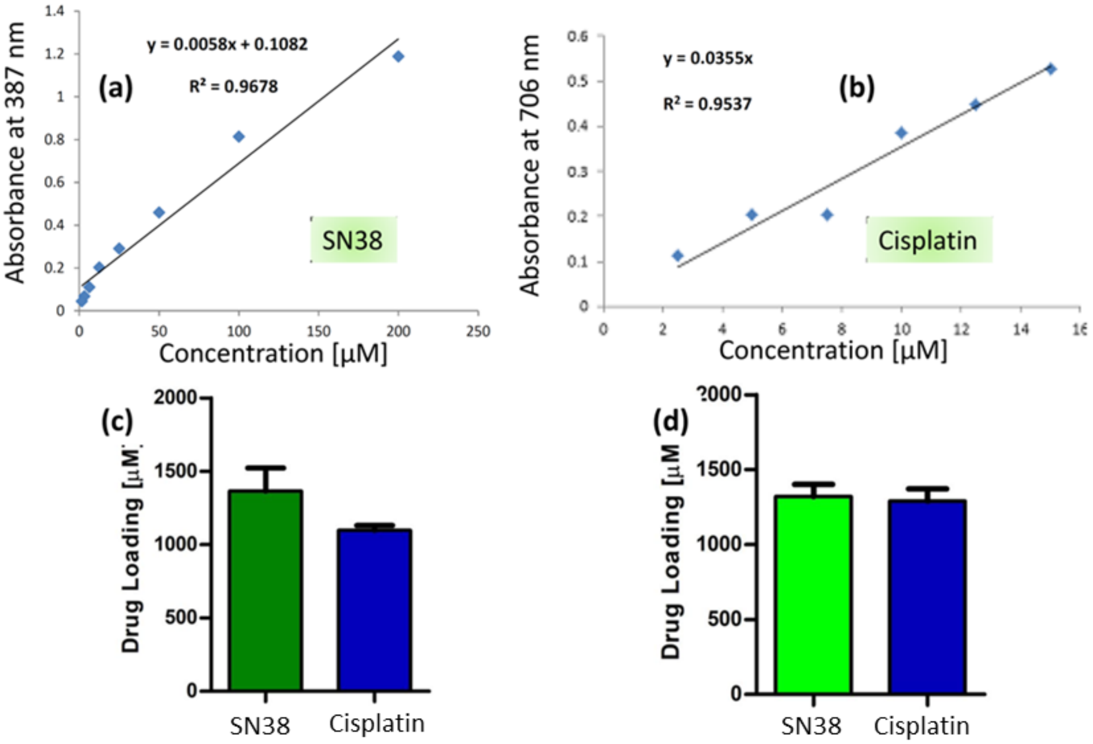

Fig. S5: (a,b) Concentration versus absorbance calibration graph of SN38 and cisplatin at characteristic wavelength respectively. (c,d) loading of SN38 and cisplatin in GO-PEG-NPs and GO-PMA-NPs respectively, calculated from the calibration graph.

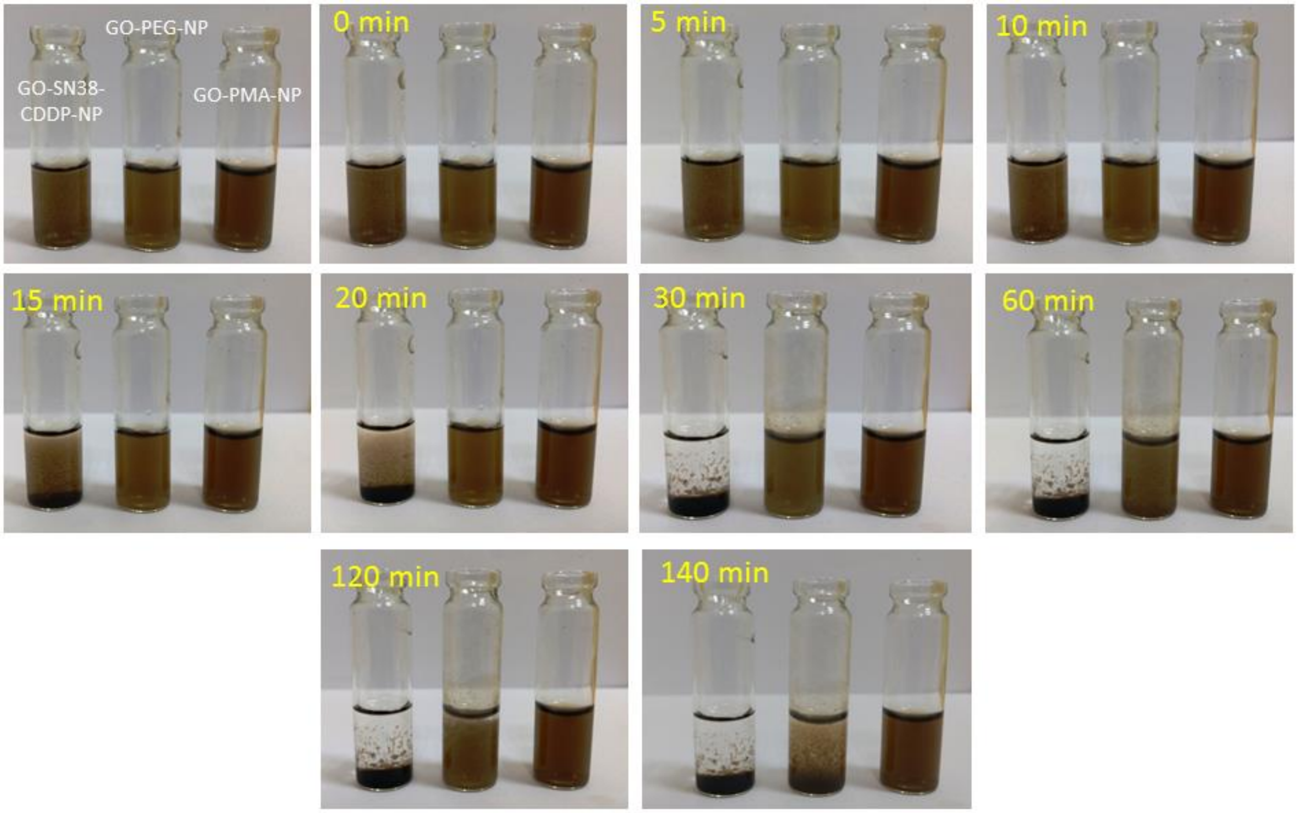

Fig. S6: Images of colloidal stability of GO-SN38-CDDP-NPs, GO-PEG-NPs and GO-PMA-NPs at different time intervals in water.

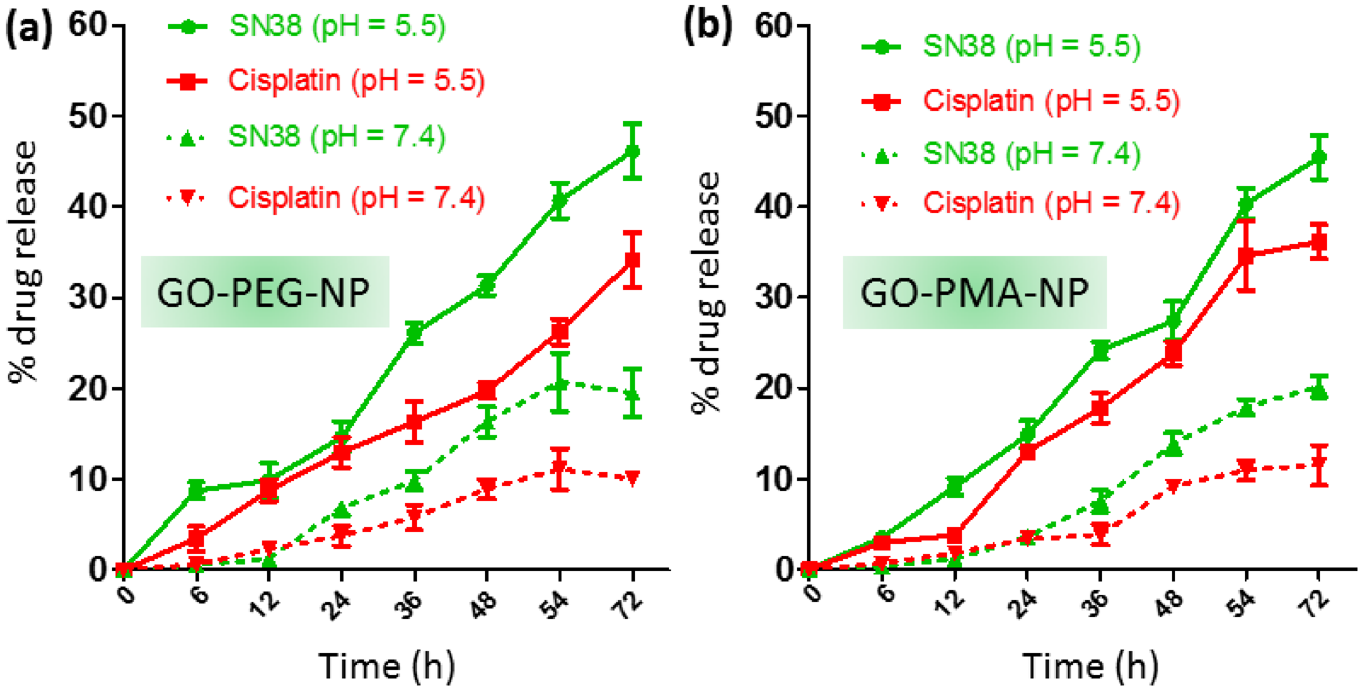

Fig. S7: (a, b) Release profile of SN38 and cisplatin at pH = 5.5 and 7.4 from GO-PEG-NPs and GO-PMA-NPs respectively.

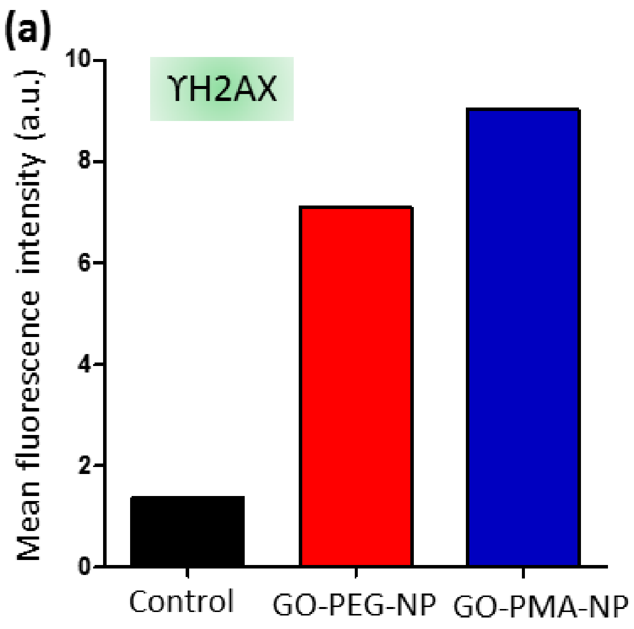

Fig. S8: (a, b) Quantification of mean fluorescence intensity from confocal microscopy in HeLa cells after treatment with GO-PEG-NPs and GO-PMA-NPs respectively for 24h followed by incubation with YH2AX and p53 primary antibodies.

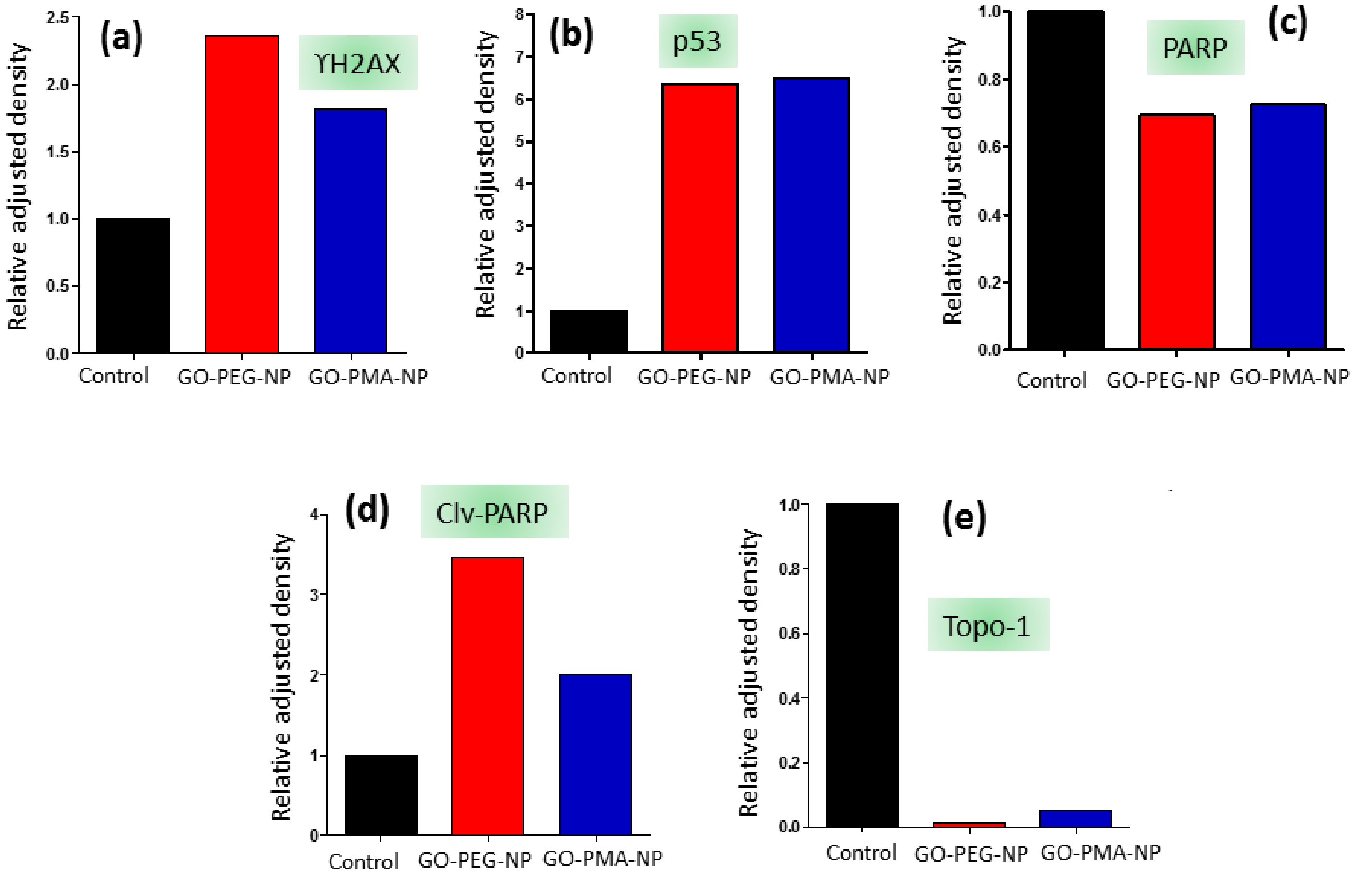

Fig. S9: (a-e) Quantification of ϒH2AX, p53, PARP, cleaved PARP and Topoisomerase-I from Western blot analysis in HeLa cells after treatment with GO-PEG-NPs and GO-PMA-NPs for 24h.

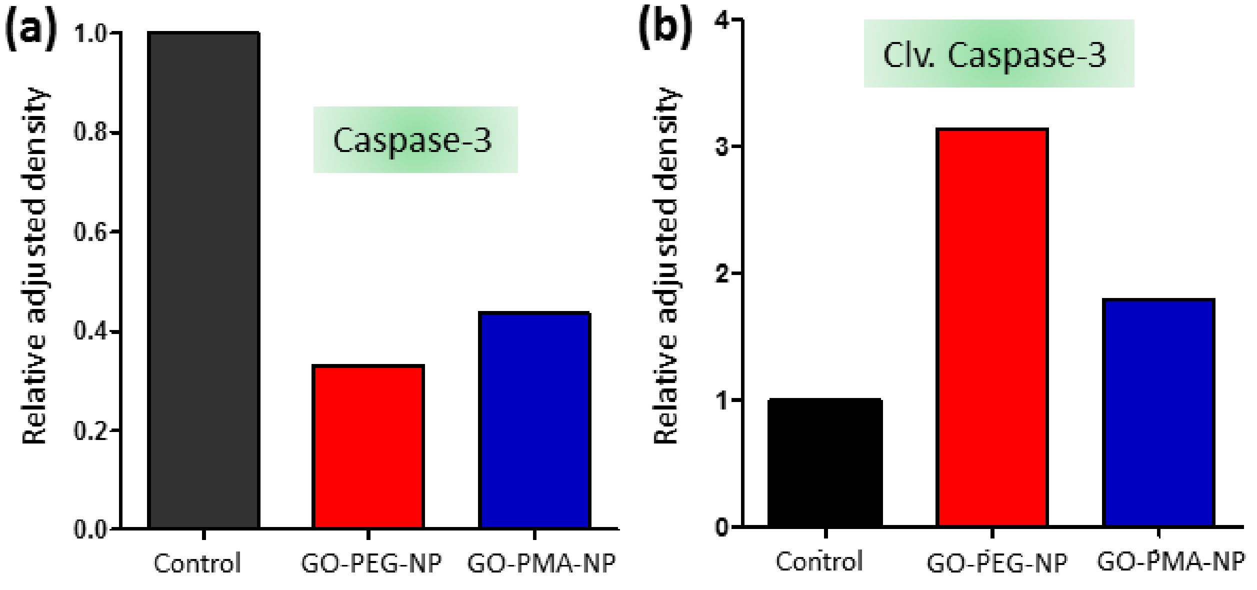

Fig. S10: (a,b) Quantification of caspase-3 and cleaved caspase-3 from Western blot analysis in HeLa cells after treatment with GO-PEG-NPs and GO-PMA-NPs for 24h.

Time	Pearson's Correlation coefficient	Mander's Coefficients TM1 Fraction of C1 overlapping C2	TM2 Fraction of C2 overlapping C1	% Volume Colocalization
1 h	0.5387	0.3028	0.3768	**15.53%**
3 h	0.625	0.5187	0.6374	**25.87%**
6 h	0.716	0.8552	0.9328	**44.53%**

Table S1: Quantification of % volume colocalization from confocal microscopy in HeLa cells after treatment with GO-PEG-NPs for 1h, 3h and 6h. Lysosomes were stained with LysoTracker Red DND 99.

Time	Pearson's Correlation coefficient	Mander's Coefficients TM1 Fraction of C1 overlapping C2	TM2 Fraction of C2 overlapping C1	% Volume Colocalization
1 h	0.364	0.3682	0.5141	**11.01%**
3 h	0.6167	0.6608	0.6201	**23.41%**
6 h	0.6956	0.8033	0.788	**37.00%**

Table S2: Quantification of % volume colocalization from confocal microscopy in HeLa cells after treatment with GO-PMA-NPs for 1h, 3h and 6h. Lysosomes were stained with LysoTracker Red DND 99.

Treatment	Pearson's Correlation coefficient	Mander's Coefficients TM1 Fraction of C1 overlapping C2	TM2 Fraction of C2 overlapping C1	% Volume Colocalization
Control	0.5227	0.6379	0.5412	**38.58%**
Chlorpromazine	0.3526	0.3307	0.3526	**11.15%**
Amiloride	0.7596	0.7753	0.777	**41.07%**
Genistein	0.6607	0.7485	0.6415	**26.49%**

Table S3: Quantification of % volume colocalization from confocal microscopy in HeLa cells after treatment with endocytosis inhibitors (chlorpromazine, amiloride and genistein) followed by GO-PEG-NPs for 2h. Lysosomes were stained with LysoTracker Red DND 99.

Treatment	Pearson's Correlation coefficient	Mander's Coefficients TM1 Fraction of C1 overlapping C2	TM2 Fraction of C2 overlapping C1	% Volume Colocalization
Control	0.7255	0.9417	0.8174	**40.64%**
Chlorpromazine	0.6134	0.6444	0.4466	**15.85%**
Amiloride	0.7775	0.7811	0.6754	**35.31%**
Genistein	0.6993	0.7314	0.6258	**31.32%**

Table S4: Quantification of % volume colocalization from confocal microscopy in HeLa cells after treatment with endocytosis inhibitors (chlorpromazine, amiloride and genistein) followed by GO-PMA-NPs for 2h. Lysosomes were stained with LysoTracker Red DND 99.

References:

1. Xu, Z.; Wang, S.; Li, Y.; Wang, M.; Shi, P.; Huang, X. Covalent Functionalization of Graphene Oxide with Biocompatible Poly (Ethylene Glycol) for Delivery of Paclitaxel. *ACS Appl. Mater. Interfaces*, **2014**, *6*, 17268–17276.

2. Liu, Z.; Robinson, J. T.; Sun, X.; Dai, H. PEGylated Nanographene Oxide for Delivery of Water-Insoluble Cancer Drugs. *J. Am. Chem. Soc.* **2008**, *130*, 10876–10877.

3. Nandi, A.; Mallick, A.; More, P.; Sengupta, P.; Ballav, N.; Basu, S. Cisplatin-Induced Self-Assembly of Graphene Oxide Sheets into Spherical Nanoparticles for Damaging Sub-Cellular DNA. *Chem. Commun.* **2017**, *53*, 1409–1412.

4. Nandi, A.; Ghosh, C.; Bajpai, A.; Basu, S. Graphene Oxide Nanocells for Impairing Topoisomerase and DNA in Cancer Cells. *J. Mater. Chem. B* **2019**,*7*, 4191-4197.
